# FAM-FACE-SG: a score for risk stratification of frequent hospital admitters

**DOI:** 10.1186/s12911-017-0441-5

**Published:** 2017-04-08

**Authors:** Lian Leng Low, Nan Liu, Kheng Hock Lee, Marcus Eng Hock Ong, Sijia Wang, Xuan Jing, Julian Thumboo

**Affiliations:** 1 Department of Family Medicine & Continuing Care, Singapore General Hospital, Singapore, Singapore; 2 Family Medicine Program, Duke-NUS Medical School, Singapore, Singapore; 3 Health Services Research Centre, Singapore Health Services, Singapore, Singapore; 4 Centre for Quantitative Medicine, Duke-NUS Medical School, Singapore, Singapore; 5 Department of Emergency Medicine, Singapore General Hospital, Singapore, Singapore; 6 Health Services and Systems Research, Duke-NUS Medical School, Singapore, Singapore; 7 Integrated Health Information Systems, Singapore, Singapore; 8 Department of Rheumatology and Immunology, Singapore General Hospital, Singapore, Singapore

**Keywords:** Risk score, Stratification, Frequent hospital admitters, Electronic health record, LACE index

## Abstract

**Background:**

An accurate risk stratification tool is critical in identifying patients who are at high risk of frequent hospital readmissions. While 30-day hospital readmissions have been widely studied, there is increasing interest in identifying potential high-cost users or frequent hospital admitters. In this study, we aimed to derive and validate a risk stratification tool to predict frequent hospital admitters.

**Methods:**

We conducted a retrospective cohort study using the readily available clinical and administrative data from the electronic health records of a tertiary hospital in Singapore. The primary outcome was chosen as three or more inpatient readmissions within 12 months of index discharge. We used univariable and multivariable logistic regression models to build a frequent hospital admission risk score (FAM-FACE-SG) by incorporating demographics, indicators of socioeconomic status, prior healthcare utilization, markers of acute illness burden and markers of chronic illness burden. We further validated the risk score on a separate dataset and compared its performance with the LACE index using the receiver operating characteristic analysis.

**Results:**

Our study included 25,244 patients, with 70% randomly selected patients for risk score derivation and the remaining 30% for validation. Overall, 4,322 patients (17.1%) met the outcome. The final FAM-FACE-SG score consisted of nine components: Furosemide (Intravenous 40 mg and above during index admission); Admissions in past one year; Medifund (Required financial assistance); Frequent emergency department (ED) use (≥3 ED visits in 6 month before index admission); Anti-depressants in past one year; Charlson comorbidity index; End Stage Renal Failure on Dialysis; Subsidized ward stay; and Geriatric patient or not. In the experiments, the FAM-FACE-SG score had good discriminative ability with an area under the curve (AUC) of 0.839 (95% confidence interval [CI]: 0.825–0.853) for risk prediction of frequent hospital admission. In comparison, the LACE index only achieved an AUC of 0.761 (0.745–0.777).

**Conclusions:**

The FAM-FACE-SG score shows strong potential for implementation to provide near real-time prediction of frequent admissions. It may serve as the first step to identify high risk patients to receive resource intensive interventions.

## Background

Health systems worldwide continue to struggle with the rapidly ageing population and increasing chronic disease burden. One key focus to reduce over reliance on hospital care is to improve the care transitions of patients at high risk of readmissions or frequent hospital admissions. An accurate, validated readmission risk stratification tool is the critical first step to identify these high risk patients to receive resource intensive interventions. To date, almost all readmission risk stratification tools have focused on predicting 30-day readmissions [1, 2]. This is in line with section 3025 of the United States Affordable Care Act that mandated the Centers for Medicare and Medicaid services to penalize hospitals with excessive readmission rates for heart failure, acute myocardial infarction and pneumonia [3]. While readmissions are arguably most preventable in the immediate post-discharge period, there is a strong case to be made for developing sustainable integrated care programs with emphasis on longer term outcomes. There is increasing interest in identifying potential high-cost users or frequent hospital admitters [4–8] who account for a disproportionate amount of healthcare spending [6, 9]. Moreover, there is significant overlap between frequent hospital admitters and 30-day re-admitters. Black et al. found that although patients with three or more readmissions accounted for only 10.2% of patients, these patients accounted for 71.6% of 30-day readmissions [10]. Lack of coordinated programs to improve the care outcomes for these high risk patients risk a vicious spiral of increasing healthcare utilization and expenditure and poorer quality of life. In heart failure patients, it is well known that each episode of hospitalization is associated with an incrementally poorer prognosis and greater mortality [11]. Efforts to reduce avoidable readmissions need to look beyond the current focus on a single hospital discharge and transition period to a patient-centric focus that manages high risk patients as a patient segment.

Singapore is a developed city state in South East Asia with a population of 5.6 million. In 2014, Bloomberg ranked the Singapore healthcare system as the most efficient in the world, despite spending a relatively small proportion of gross domestic product on healthcare and having one of the lowest healthcare costs per capita [12]. Like most developed health systems struggling with a rapidly ageing population, increasing chronic disease burden and a shrinking workforce, the Singapore healthcare system has been transforming from a hospital centric model to a population centric model to avoid an unsustainable healthcare system. To achieve this, the healthcare system has been re-organized into regional health systems (RHS) that aimed to deliver value-based, patient centered care [8]. In each RHS, there is a primary tertiary hospital as well as a secondary hospital that provides intermediate and rehabilitation care; in its region, there are primary care and long-term care services to link vertical and horizontal integration of care.

Since 2015, each RHS receives capitated funding to improve the care transitions and reduce readmission risk of patients at high risk of frequent admissions in its catchment population. Frequent admitters or “familiar faces” in Singapore are defined as patients with three or more inpatient admissions in a year. These are high-cost patients, with an average cost per patient approaching SGD 30,000 a year [7]. In the SingHealth RHS, 80 senior nurses have been trained in transitional care as patient navigators [13] (PNs) to perform case management. Transitional care services have been re-organized into integrated practice units [14, 15] to coordinate the care of patients through the complex healthcare system for the one year after hospital discharge. These interventions include a comprehensive needs assessment, followed by case management, post-discharge monitoring [15] and home care [14]. These interventions are resource intensive and must be targeted at patients at highest risk of frequent admissions to be cost-effective and sustainable. An accurate and validated frequent hospital admission predictive risk score is critical for risk stratification. The risk score should ideally be automated for easy adoption by healthcare users and provide real-time/near real-time risk scores early in the admission in order to impact interventions before hospital discharge.

SingHealth RHS has a well-established electronic health record (EHR) system that was developed and implemented more than 10 years ago. The EHR integrates information from multiple sources including administrative data (for example, patient demographics), clinical data and ancillary, called the Electronic Health Intelligence System [16]. The Singapore system is comparable with the definition of EHR used in the US or Europe. On a daily basis, data from these multiple sources are extracted, transformed and loaded onto the enterprise data warehouse. Further use can be in the form of reports, dashboards and mobile applications to support business and operational needs. Leveraging on this well-established EHR system, it is possible to derive a near real-time (refreshed on a daily basis) automated predictive risk score to identify high risk patients for intervention. Currently, based on Kansagara’s review [1] and more recent Zhou’s review [17], around ten published predictive models utilized real-time administrative data.

Our study aims to address the lack of an accurate, validated frequent admitter risk score. Our primary objective is to derive a real-time frequent hospital admission risk stratification model to predict frequent hospital admitters or “familiar faces in Singapore”. We will compare our model with the LACE index [18], an established risk stratification tool. The LACE index includes four components, namely length of stay (“L”), acuity of the admission (“A”), comorbidity of the patient (“C”) and emergency department use (“E”). It can be easily calculated and has been widely used for predicting the risk of readmissions within 30 days after discharge of hospital. However, the LACE index demonstrated inconsistent performance [17]. Our study hypothesizes that by including potentially discriminatory predictors such as medications used during the hospitalization episode and indicators of socioeconomic status, the new risk stratification model will perform better than the LACE index at predicting frequent hospital admissions within 12 months of discharge.

## Methods

### Study design and participants

We conducted a retrospective observational cohort study using the EHR data of the Singapore General Hospital (SGH). This study was approved by SingHealth Centralized Institutional Review Board (CIRB 2015/2696) with waiver of informed consent. We included all admitted adult patients (≥21 years of age, i.e., the legal age for majority in Singapore) from January 1, 2013 to May 31, 2014. For patients who were admitted multiple times, we only included the index admission. We excluded patients who were non-residents, died during the index admission, and whose admission specialty was obstetrics, emergency medicine, dentistry or ophthalmology. Emergency medicine admissions were excluded as these were observation ward admissions rather than true hospital admissions. Patients admitted to the emergency medicine observation ward are typically monitored up to 24 h and subsequently discharged home or converted to hospital admissions according to their clinical circumstances. Patients converted to hospital admissions would be captured in our dataset. We also excluded patients admitted to obstetrics as these admissions are pregnancy related; and admissions to dentistry and ophthalmology which are usually elective in nature.

### Outcome and variables

The primary outcome in this study was chosen as three or more inpatient readmissions within 12 months of discharge. Candidate variables were selected by a group of clinical experts based on our preliminary study on frequent admissions [2] and existing literature [1, 16, 19, 20]. Our independent variables included patient demographics (age, gender and ethnicity), comorbidities, healthcare utilization in preceding year (number of admissions, number of specialist outpatient clinic visits, and number of emergency department visits), and variables reflecting socioeconomic status. Specifically, comorbidities (the Charlson and Elixhauser Comorbidity Index measures [21–23]) were identified using International Classification of Diseases Ninth and Tenth Revision (ICD-9 and ICD-10) codes for major diagnoses in the past seven years (as shown in Table 1). Our approach is among the most comprehensive in the literature [24] and would reduce potential lapses in diagnostic coding.

**Table 1:** Baseline characteristics of the patient cohort

	Model Derivation Dataset			Model Validation Dataset		
Variable	All patients (*n* = 17670)	Frequent admitters (*n* = 3061)	Non frequent admitters (*n* = 14609)	All patients (*n* = 7574)	Frequent admitters (*n* = 1261)	Non frequent admitters (*n* = 6313)
Patient Demographics						
Age, Mean (SD)	58.6 (17.92)	65.15 (15.15)	57.26 (18.14)	58.3 (17.93)	64.53 (15.17)	57.06 (18.18)
Gender, Male (%)	9127 (51.6%)	1579 (52.6%)	7548 (51.5%)	3929 (51.9%)	680 (53.9%)	3249 (51.5%)
Ethnicity						
Chinese (%)	12772 (72.3%)	2234 (74.4%)	10538 (71.8%)	5417 (71.5%)	919 (72.9%)	4498 (71.3%)
Indian (%)	1745 (9.9%)	281 (9.4%)	1464 (10%)	776 (10.2%)	123 (9.8%)	653 (10.3%)
Malay (%)	2089 (11.8%)	394 (13.1%)	1695 (11.6%)	924 (12.2%)	179 (14.2%)	745 (11.8%)
Others (%)	1065 (6%)	93 (3.1%)	972 (6.6%)	456 (6%)	40 (3.2%)	416 (6.6%)
Indicators of Socioeconomic Status						
Required financial assistance using Medifund (%)	248 (1.4%)	86 (2.9%)	162 (1.1%)	102 (1.3%)	35 (2.8%)	67 (1.1%)
Stayed in a subsidized ward during index admission (%)	13487 (76.3%)	2688 (89.5%)	10799 (73.6%)	5807 (76.7%)	1140 (90.4%)	4667 (73.9%)
Past Healthcare Utilization						
ED visits (6 month before index admission), Mean (SD)	0.37 (0.91)	0.67 (1.55)	0.31 (0.69)	0.36 (0.92)	0.69 (1.64)	0.3 (0.68)
Specialist Clinic visits (1 year before index admission), Mean (SD)	2.24 (5.38)	4.25 (7.68)	1.83 (4.67)	2.14 (5.28)	4.16 (7.74)	1.74 (4.52)
Hospital admissions (1 year before index admission), Mean (SD)	0.33 (1.02)	0.95 (1.83)	0.21 (0.7)	0.34 (1.14)	1.04 (2.2)	0.2 (0.68)
Markers of Acute Illness Burden						
Index admission was urgent (%)	13501 (76.4%)	2450 (81.6%)	11051 (75.3%)	5764 (76.1%)	992 (78.7%)	4772 (75.6%)
Required second line antibiotics during index admission (%)	860 (4.9%)	202 (6.7%)	658 (4.5%)	349 (4.6%)	80 (6.3%)	269 (4.3%)
Required inpatient dialysis during index admission (%)	580 (3.3%)	290 (9.7%)	290 (2%)	256 (3.4%)	126 (10%)	130 (2.1%)
Required intravenous Furosemide 40 mg and above during index admission (%)	1038 (5.9%)	355 (11.8%)	683 (4.7%)	435 (5.7%)	143 (11.3%)	292 (4.6%)
Required isolation during index admission (%)	216 (1.2%)	54 (1.8%)	162 (1.1%)	98 (1.3%)	21 (1.7%)	77 (1.2%)
Length of stay of index admission, Mean (SD)	5.54 (11.16)	7.49 (10.88)	5.14 (11.17)	5.55 (12.29)	7.56 (11.82)	5.15 (12.35)
Charlson Comorbidity Index^a^, Mean (SD)	1.51 (2.44)	3.62 (3.03)	1.08 (2.05)	1.45 (2.39)	3.56 (3)	1.03 (2)
Medical Comorbidities^b^						
Stroke	291 (1.6%)	49 (1.6%)	242 (1.6%)	124 (1.6%)	20 (1.6%)	104 (1.6%)
Metastatic Disease	1924 (10.9%)	349 (11.6%)	1575 (10.7%)	831 (11%)	156 (12.4%)	675 (10.7%)
Non-metastatic malignancy	3116 (17.6%)	530 (17.7%)	2586 (17.6%)	1352 (17.9%)	237 (18.8%)	1115 (17.7%)
Peripheral Vascular Disease	717 (4.1%)	123 (4.1%)	594 (4%)	321 (4.2%)	48 (3.8%)	273 (4.3%)
Heart Failure or Fluid Overload	2146 (12.1%)	392 (13.1%)	1754 (12%)	937 (12.4%)	140 (11.1%)	797 (12.6%)
Pressure Ulcer	583 (3.3%)	106 (3.5%)	477 (3.3%)	259 (3.4%)	45 (3.6%)	214 (3.4%)
Thromboembolism	1141 (6.5%)	224 (7.5%)	917 (6.3%)	510 (6.7%)	91 (7.2%)	419 (6.6%)
Spine Fracture	488 (2.8%)	92 (3.1%)	396 (2.7%)	198 (2.6%)	39 (3.1%)	159 (2.5%)
Coronary Heart Disease or Myocardial Infarction	2624 (14.8%)	504 (16.8%)	2120 (14.5%)	1151 (15.2%)	199 (15.8%)	952 (15.1%)
Hip Fracture	375 (2.1%)	60 (2%)	315 (2.1%)	144 (1.9%)	28 (2.2%)	116 (1.8%)
Atrial Fibrillation	1348 (7.6%)	256 (8.5%)	1092 (7.4%)	612 (8.1%)	98 (7.8%)	514 (8.1%)
Epilepsy	180 (1%)	31 (1%)	149 (1%)	79 (1%)	11 (0.9%)	68 (1.1%)
Parkinsonism	284 (1.6%)	60 (2%)	224 (1.5%)	124 (1.6%)	28 (2.2%)	96 (1.5%)
Anxiety	170 (1%)	23 (0.8%)	147 (1%)	82 (1.1%)	12 (1%)	70 (1.1%)
Bipolar Disorder	48 (0.3%)	9 (0.3%)	39 (0.3%)	24 (0.3%)	3 (0.2%)	21 (0.3%)
Collagen Vascular Disease	270 (1.5%)	40 (1.3%)	230 (1.6%)	107 (1.4%)	14 (1.1%)	93 (1.5%)
Dementia	616 (3.5%)	99 (3.3%)	517 (3.5%)	289 (3.8%)	49 (3.9%)	240 (3.8%)
Hypothyroidism	406 (2.3%)	62 (2.1%)	344 (2.3%)	183 (2.4%)	26 (2.1%)	157 (2.5%)
Chronic Kidney Disease, Stages 1–4	3075 (17.4%)	554 (18.5%)	2521 (17.2%)	1318 (17.4%)	217 (17.2%)	1101 (17.4%)
Chronic Obstructive Pulmonary Disease	503 (2.8%)	86 (2.9%)	417 (2.8%)	213 (2.8%)	26 (2.1%)	187 (3%)
Osteoarthritis	1499 (8.5%)	247 (8.2%)	1252 (8.5%)	657 (8.7%)	101 (8%)	556 (8.8%)
Benign Prostatic Hypertrophy	856 (4.8%)	138 (4.6%)	718 (4.9%)	373 (4.9%)	59 (4.7%)	314 (5%)
Asthma	536 (3%)	99 (3.3%)	437 (3%)	212 (2.8%)	33 (2.6%)	179 (2.8%)
Hyperlipidemia	5026 (28.4%)	900 (30%)	4126 (28.1%)	2186 (28.9%)	366 (29%)	1820 (28.8%)
Hypertension	7051 (39.9%)	1240 (41.3%)	5811 (39.6%)	3029 (40%)	493 (39.1%)	2536 (40.2%)
Chronic Kidney Disease Stage 5 or End Stage Renal Failure	2384 (13.5%)	441 (14.7%)	1943 (13.2%)	977 (12.9%)	150 (11.9%)	827 (13.1%)
Diabetes	4388 (24.8%)	779 (25.9%)	3609 (24.6%)	1908 (25.2%)	319 (25.3%)	1589 (25.2%)
History of Alcoholism	297 (1.7%)	60 (2%)	237 (1.6%)	130 (1.7%)	30 (2.4%)	100 (1.6%)
Treatment with anti-depressants^c^	1753 (9.9%)	706 (23.5%)	1047 (7.1%)	726 (9.6%)	298 (23.6%)	428 (6.8%)

*FA* frequent admitters

^a^Based on ICD codes of index admission

^b^Based on ICD codes in the preceding seven years

^c^Based on discharge and outpatient prescriptions in the preceding one year

To assess the socioeconomic status, we used two indicators: whether the patient stayed in subsidized ward class during index admission and whether the patient was assisted by Medifund. In Singapore, hospitals use means testing to evaluate the patient’s eligibility for subsidized wards. It is based on patients’ income or the annual value of their residence. The government aims to share the limited subsidies to all Singaporeans in a fair manner by providing more subsidies to people with lower socioeconomic status. Additionally, those patients who are still unable to afford their healthcare bills after receiving government subsidies and other means of payments including insurance may apply for Medifund assistance. Medifund is a safety net uniquely provided by Singapore government to help patients with special financial difficulties [25]. The actual amount of assistance also depends on the patient’s socioeconomic status.

We selected seven variables of acute illness burden potentially predictive of frequent admissions. As listed in Table 1, these variables included whether the index admission was urgent, whether second line antibiotics were required, whether treatment with intravenous furosemide 40 mg or more was required, whether inpatient dialysis was required, length of stay of the index admission, and the Charlson Comorbidity Index (CCI) of index admission. One of the novelties in this study was to use the CCI in weighting the index discharge diagnoses, which was already a key marker for mortality within one year [22]. Intravenous furosemide was chosen as one of candidate variables because it has been used in acute treatment of heart failure and therefore a proxy marker of heart disease severity. Moreover, we found it a significant predictor of frequent hospital admission risk [2] and 30-day readmission risk [26].

Antidepressants play an important role in the treatment of depression and may be associated with hospital readmissions [27, 28]. We hypothesized that the use of antidepressants would affect the risk of frequent admissions. Patients with depression are more likely treated as outpatients and would not have discharge ICD codes for depression. Therefore, we included the variable of whether the patient had treatment with antidepressants in the past one year in our list of predictors.

### Model derivation and validation

We randomly selected 70% of the data for model derivation and the remaining 30% of the data for model validation. Due to low prevalence rate of frequent hospital admitters, we split the data by strata, that is, we randomly selected equal percentage of data from both frequent admitters group and non-frequent admitters group into both derivation and validation sets. We conducted univariable and multivariable logistic regression to measure the independent association of predictive variables with hospital frequent admission. We chose the most significant (*p* < 0.02) predictive variables and used clinical knowledge to determine a set of candidate variables for final model derivation. We adopted a popular risk score derivation method [18, 29] to convert the final logistic model into a risk index. The principle to derive the risk point for each variable was to divide its regression β coefficient by the smallest β coefficients of all variables, and to round the value to the nearest whole number. Then, the final score was calculated by summing up the risk points of all variables.

We assessed model discrimination using the validation set, that is, randomly selected 30% of the data. We conducted the receiver operating characteristic (ROC) analysis to evaluate the performance of the predictive model. From the ROC analysis, we also computed measures of diagnostic accuracy such as sensitivity, specificity, positive predictive value (PPV), and negative predictive value (NPV). We performed all analyses using R version 3.2.3 (R Foundation, Vienna, Austria).

## Results

**Fig. 1:**
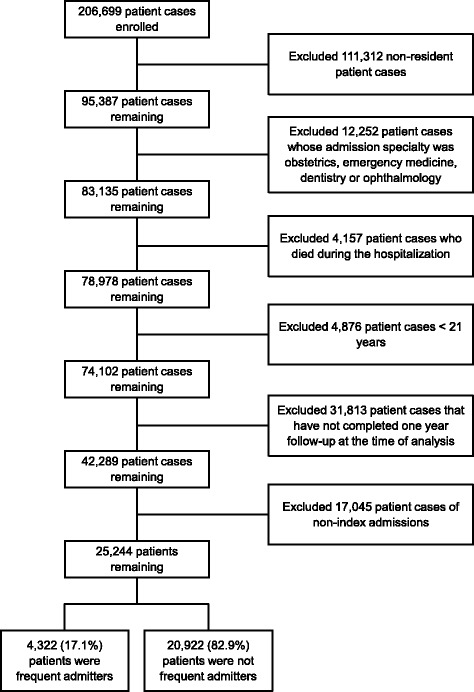
Patient selection in the study

From January 1, 2013 to May 31, 2014, a total of 206,699 patient admissions were recorded in SGH. Among all records, we excluded 111,312 non-resident patient cases, 12,252 patient cases whose admission specialty was obstetrics, emergency medicine, dentistry or ophthalmology, and 4,157 patient cases who died during the hospitalization. With the remaining 74,102 adult patient cases, we further excluded 31,813 patient cases that have not completed one year follow-up at the time of analysis and 17,045 cases of non-index admissions. Overall, 25,244 patients were included in this study. Of these, 4,322 patients (17.1%) had three or more admissions in the one year period following index discharge (Fig. 1). These patients were classified as frequent admitters according to our definition. The final model contained nine significant predictors (Furosemide intravenous 40 mg and above; Admissions in the past one year; Medifund; Frequent Emergency Department use ≥3 in the past six months; Anti-depressants in past one year; Charlson Comorbidity Index; End stage renal failure on dialysis; Subsidized ward stay; and Geriatric patient). Accordingly, we termed the novel index as the FAM-FACE-SG score.

Table 1 tabulates the baseline characteristics of the study population. The mean age of frequent admitters was 65 (standard deviation [SD] = 15) years and the mean age of non-frequent admitters was 57 (SD = 18) years. Male patients accounted for 51.7% of the total study population. Of all index admissions, 76.3% (*n* = 19,265) were urgent. The mean length of stay of index admission was 5.54 days. On average, frequent admitters had a mean CCI of 3.60 and non-frequent admitters had a mean CCI of 1.06. Compared to other patients, frequently admitted patients were more likely to be older, required a significantly longer stay for their index admission, had a higher CCI score, and had more ED admissions in the preceding six months.

The results of univariable and multivariable logistic regression analysis on frequent hospital admissions are presented in Table 2. Nine variables were found to be significantly associated with frequent hospital admissions. As shown in Table 3, these variables included age, required financial assistance using Medifund, stayed in a subsidized ward class during index admission, number of ED visits within the past six months before index admission, number of hospital admissions within the past one year before index admission, required dialysis during index admission, treatment with intravenous Furosemide 40 mg and above during index admission, Charlson Comorbidity Index, and treatment with antidepressants in the past one year. The top two strongest independent predictors were admission to a subsidized ward class during index admission (adjusted OR: 4.05, 95% confidence interval [CI]: 2.54–6.85) and treatment with antidepressants in the past one year (adjusted odds ratio [OR]: 2.33, 95% CI: 2.06–2.64). Additionally, patients who required financial assistance using Medifund were 1.49 times more likely (adjusted OR: 1.49, 95% CI: 1.08–2.04) to become a FA. Every increase of one point on the CCI score was associated with 31% increased risk of becoming a FA (adjusted OR: 1.31, 95% CI: 1.28–1.33).

**Table 2:** Univariable and multivariable logistic regression

Variable	Unadjusted OR (95% CI)	*p*-value	Adjusted OR (95% CI)	*p*-value*
Patient Demographics				
Age	1.03 (1.02, 1.03)	<0.001	1.01 (1.01, 1.017)	<0.001^†^
Gender (Male)	1.05 (0.97, 1.13)	0.254	1.02 (0.93, 1.12)	0.671
Ethnicity				
Others	Baseline			
Chinese	1.14 (1.04, 1.25)	0.004	0.90 (0.70, 1.16)	0.407
Indian	0.93 (0.81, 1.06)	0.300	1.18 (0.89, 1.59)	0.249
Malay	1.16 (1.03, 1.30)	0.015	1.04 (0.79, 1.39)	0.764
Indicators of Socioeconomic Status				
Required financial assistance using Medifund	2.64 (2.02, 3.43)	<0.001	1.49 (1.08, 2.04)	0.015^†^
Stayed in a subsidized ward during index admission	3.07 (2.72, 3.47)	<0.001	4.05 (2.54, 6.85)	<0.001^†^
Past Healthcare Utilization				
ED visits (6 month before index admission)	1.47 (1.41,1.53)	<0.001	1.15 (1.09, 1.22)	<0.001^†^
Specialist Clinic visits (1 year before index admission)	1.07 (1.06, 1.07)	<0.001	1.01 (1.00, 1.02)	0.088
Hospital admissions (1 year before index admission)	1.77 (1.70, 1.84)	<0.001	1.32 (1.26, 1.39)	<0.001^†^
Markers of Acute Illness Burden				
Index admission was urgent	1.45 (1.32, 1.61)	<0.001	0.92 (0.73, 1.16)	0.465
Required second line antibiotics during index admission	1.54 (1.30, 1.80)	<0.001	1.07 (0.86, 1.32)	0.534
Required inpatient dialysis during index admission	5.30 (4.48, 6.27)	<0.001	1.72 (1.38, 2.15)	<0.001^†^
Required intravenous Furosemide 40 mg and above during index admission	2.75 (2.40, 3.14)	<0.001	1.26 (1.07, 1.48)	0.005^†^
Required isolation during index admission	1.64 (1.19, 2.22)	0.002	1.19 (0.81, 1.72)	0.375
Length of stay of index admission	1.02 (1.01, 1.02)	<0.001	1.00 (1.00, 1.00)	0.919
Charlson Comorbidity Index^a^	1.41 (1.38, 1.42)	<0.001	1.31 (1.28, 1.33)	<0.001^†^
Medical Comorbidities^b^				
Stroke	0.99 (0.72, 1.34)	0.945	0.93 (0.64, 1.34)	0.712
Metastatic Disease	1.09 (0.97, 1.24)	0.154	1.07 (0.90, 1.28)	0.43
Non-metastatic malignancy	1.00 (0.90, 1.11)	0.973	0.96 (0.83, 1.11)	0.569
Peripheral Vascular Disease	1.01 (0.83, 1.23)	0.904	1.10 (0.86, 1.40)	0.456
Heart Failure or Fluid Overload	1.11 (0.98, 1.24)	0.093	1.00 (0.84,1.19)	0.978
Pressure Ulcer	1.09 (0.88, 1.34)	0.435	1.14 (0.88,1.48)	0.307
Thromboembolism	1.21 (1.04, 1.41)	0.014	1.21 (1.00,1.46)	0.045
Spine Fracture	1.14 (0.90, 1.43)	0.266	1.05 (0.79, 1.38)	0.743
Coronary Heart Disease or Myocardial Infarction	1.19 (1.07, 1.33)	0.001	1.1 (0.94, 1.29)	0.225
Hip Fracture	0.93 (0.70, 1.22)	0.607	0.96 (0.69, 1.32)	0.795
Atrial Fibrillation	1.16 (1.00, 1.33)	0.042	1.1 (0.92, 1.32)	0.302
Epilepsy	1.02 (0.68, 1.48)	0.933	0.82 (0.51, 1.28)	0.394
Parkinsonism	1.32 (0.98, 1.74)	0.062	1.43 (1.01, 2.00)	0.043
Anxiety	0.76 (0.48, 1.16)	0.229	0.57 (0.33, 0.92)	0.028
Bipolar Disorder	1.13 (0.51, 2.23)	0.745	1.18 (0.48, 2.65)	0.699
Collagen Vascular Disease	0.85 (0.60, 1.17)	0.338	0.76 (0.51, 1.12)	0.175
Dementia	0.93 (0.75, 1.16)	0.537	0.91 (0.70, 1.18)	0.498
Hypothyroidism	0.88 (0.66, 1.15)	0.352	0.93 (0.67, 1.28)	0.681
Chronic Kidney Disease, Stages 1–4	1.09 (0.98, 1.21)	0.095	0.97 (0.79, 1.18)	0.731
Chronic Obstructive Pulmonary Disease	1.01 (0.79, 1.27)	0.947	1.07 (0.80,1.41)	0.648
Osteoarthritis	0.96 (0.83,1.11)	0.582	0.90 (0.75, 1.07)	0.223
Benign Prostatic Hypertrophy	0.94 (0.77, 1.12)	0.489	0.95 (0.76,1.18)	0.619
Asthma	1.11 (0.89, 1.38)	0.354	1.22 (0.93, 1.59)	0.152
Hyperlipidemia	1.09 (1.00, 1.19)	0.04	0.99 (0.85, 1.14)	0.868
Hypertension	1.07 (0.99, 1.16)	0.085	0.96 (0.83, 1.11)	0.587
Chronic Kidney Disease Stage 5 or End Stage Renal Failure	1.13 (1.01, 1.26)	0.035	1.03 (0.83, 1.28)	0.81
Diabetes	1.07 (0.98, 1.17)	0.12	1.03 (0.90, 1.17)	0.71
History of Alcoholism	1.24 (0.93, 1.64)	0.138	0.91 (0.64, 1.27)	0.582
Treatment with anti-depressants^c^	4.00 (3.60, 4.44)	<0.001	2.33 (2.06, 2.64)	<0.001^†^

*OR* odds ratio, *ED* emergency department, *ICD* international classification of diseases

*Statistical significance was set as *p* < 0.02

^†^Variables selected for building final logistic regression model

^a^Based on ICD codes of index admission

^b^Based on ICD codes in the preceding seven years

^c^Based on discharge and outpatient prescriptions in the preceding one year

**Table 3:** Predictors for deriving the FAM-FACE-SG score

Predictor	Value	OR (95% CI)	*p*-value	β Coefficients	Normalized β Coefficients	Final Score
Furosemide (Intravenous 40 mg and above during index admission)	Yes	1.26 (1.09,1.47)	0.002	0.2311	1.8912	2
Admissions in past one year	0			–	–	0
	1–2	1.87 (1.66,2.10)	<0.001	0.6259	5.1219	5
	3–4	3.5 (2.79,4.40)	<0.001	1.2528	10.252	10
	>4	5.31 (3.82,7.45)	<0.001	1.6696	13.6628	14
Medifund (Required financial assistance)	Yes	1.7 (1.25,2.30)	0.001	0.5306	4.3421	4
Frequent ED use (≥3 ED visits in 6 month before index admission)	Yes	1.59 (1.22,2.07)	0.001	0.4637	3.7946	4
Anti-depressants in past one year	Yes	2.42 (2.14,2.73)	<0.001	0.8838	7.2324	7
Charlson Comorbidity Index	0			–	–	0
	1	2.5 (2.11,2.95)	<0.001	0.9163	7.4984	7
	2	5.11 (4.48,5.83)	<0.001	1.6312	13.3486	13
	3	7.86 (6.55,9.42)	<0.001	2.0618	16.8723	17
	≥4	11.75 (10.37,13.33)	<0.001	2.4639	20.1628	20
End Stage Renal Failure on Dialysis	Yes	1.65 (1.36,2.00)	<0.001	0.5008	4.0982	4
Subsidized ward stay	Yes	1.5 (1.31,1.71)	<0.001	0.4055	3.3183	3
Geriatric patient (Age)	<65			–	–	0
	65–84	1.13 (1.03,1.25)	0.013	0.1222	1	1
	≥85	1.43 (1.21,1.68)	<0.001	0.3577	2.9272	3

**Fig. 2:**
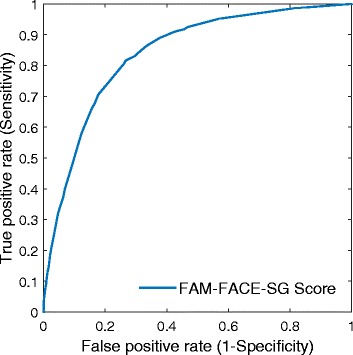
The receiver operating characteristic curve for the FAM-FACE-SG score

Figure 2 shows the ROC curve of the final logistic regression model. Using the proposed FAM-FACE-SG score, the final predictive model had better discriminative ability than the traditional LACE index (area under the curve [AUC]: 0.839, 95% CI: 0.825–0.853). The optimal cut-off for the regression model was 13, which achieved a sensitivity of 81.6% (95% CI: 79.5–83.7%) and a specificity of 73.3% (72.2–74.4%). In addition, Table 4 shows the sensitivity, specificity, PPV and NPV according to these cut-offs.

**Table 4:** Discriminatory values for FAM-FACE-SG score and the LACE index

	FAM-FACE-SG	LACE
AUC (95% CI)	0.839 (0.825–0.853)	0.761 (0.745–0.777)
Cutoff score	13	8
Sensitivity (95% CI)	81.6% (79.5%–83.7%)	65.8% (63.2%–68.4%)
Specificity (95% CI)	73.3% (72.2%–74.4%)	76.2% (75.2%–77.3%)
PPV (95% CI)	37.9% (36.1%–39.8%)	36.3% (34.3%–38.2%)
NPV (95% CI)	95.2% (94.6%–95.8%)	91.5% (90.8%–92.3%)

*AUC* area under the curve, *CI* confidence interval, *PPV* positive predictive value, *NPV* negative predictive value

## Discussion

In this study, we proposed a risk stratification model (FAM-FACE-SG) by incorporating demographics, indicators of socioeconomic status, prior healthcare utilization, markers of acute illness burden and markers of chronic illness burden for the prediction of frequent hospital admission risk in Singapore. In model validation, FAM-FACE-SG significantly outperformed the LACE index in terms of AUC (0.839 vs 0.761, *p* < 0.001). In addition, FAM-FACE-SG performs better than the LACE index in achieving higher sensitivity, specificity, positive predictive value, and negative predictive value. The LACE index had functioned as a benchmark for predicting 30-day readmission risk due to its popularity and simplicity in computation. The FAM-FACE-SG score overcomes the disadvantages of the LACE index with its near real-time availability early in the admission.

This study is novel by being the first study to derive and internally validate a risk score for frequent hospital admission risk. We considered well established predictors for readmission risk such as demographics, indicators of socioeconomic status, and prior healthcare utilization [1, 8, 30] in deriving the score. Developed in close collaboration between data scientists and clinicians who are involved in transitional care of patients, the score also incorporated clinical variables such as the use of anti-depressants as a proxy measure of clinically significant depression. We believe that beyond improving predictive performance of the tool, such variables may help us identify patients who are more amenable to clinical interventions. In addition, we incorporated markers of acute illness burden such as intravenous furosemide and dialysis into FAM-FACE-SG risk score for near real-time prediction. Our work highlights the excellent opportunity to leverage on the technological advancements in EHR and business analytics tools to generate real-time insights that support clinical care. In constructing the FAM-FACE-SG score, Charlson comorbidity index and admissions in the past one year contribute the greatest weight to the final score. While it would be ideal to achieve parsimony of the risk score by excluding Medifund and Subsidized ward stay, placed in the national context of deriving a risk stratification score to identify high risk patients as a critical first step in transitional care programs, we believe it is important to report the impact of indicators of socioeconomic status on frequent hospital admission risk.

Risk stratification enables the identification of individuals at risk of an unwanted outcome and effect targeted interventions for optimal results. Most transitional care interventions are aimed at preventing re-admissions and reducing inappropriate utilization of expensive hospital services. The optimization of transitional care requires the re-organization of care processes. Sometimes additional resources such as case managers are required. Improving outcome through transitional care must therefore be achieved at equal or lower cost of care. A clinically reliable tool that can guide the intervention towards patients who are at highest risk of suffering from sub-optimal care transition is therefore key to making such programs cost effective. The quest for a good risk prediction tool for transitional care requires it to be low cost, technically accurate and clinically practical. Tools that use data that is not routinely captured during usual care of patients will add cost and increase the administrative burden of healthcare workers. Our tool was derived using administrative and clinical data that is routinely captured during care of hospitalized patients. This averted the need for additional cost and the work of data collection. It also makes the use of the tool sustainable as it requires minimal technical support.

The key to driving implementation and adoption is automation. In deriving the FAM-FACE-SG score, we ensured that all variables can be retrieved from our EHR system near real-time to be automated into a risk score that is clinically useful to clinicians and case managers. Relevant facts and dimensions are compiled using stored procedures in our enterprise data mart before the risk score is tabulated using compiled tables. The data mart system is then scheduled to run every midnight & produce risk scores for patients admitted that same day. The risk scores are available “near real-time” to clinicians and case managers on the day after the admission day.

### Limitations

We believe that the FAM-FACE-SG score is a practical risk score that has implementation potential in other health systems. However, there are limitations to our study. First, variables in our dataset are restricted to those routinely collected in our administrative and clinical database. We did not include predictors such as caregiver availability and function which would require additional effort in data collection and therefore impractical for incorporation into a risk score. Second, we did not exclude patients who might have deceased after index hospital discharge. This may have introduced biases in model building. In fact, end of life patients who are identified as high risk for frequent hospital admission can be appropriately referred to hospice programs to reduce their readmission risk and morbidity from unnecessary hospital procedures. Third, unavailability of the required variables in calculating the FAM-FACE-SG score may prevent the use of the score in other healthcare systems. However, the method of score derivation could be adapted to customize the score in different systems.

## Conclusions

We derived a frequent hospital admission risk score (FAM-FACE-SG) incorporating demographics, indicators of socioeconomic status, prior healthcare utilization, markers of acute illness burden and markers of chronic illness burden. The FAM-FACE-SG score had excellent discriminative ability with an AUC of 0.839 for prediction of frequent hospital admission risk. It has strong potential for implementation to provide near real-time prediction. A feasibility study is required to study its effectiveness to reduce hospital admissions and external validation is necessary to ensure reproducibility in other health systems.

## Abbreviations

AUC: Area under the curve
CCI: Charlson comorbidity index
CI: Confidence interval
CIRB: Centralized institutional review board
ED: Emergency department
EHR: Electronic health record
ICD: International classification of diseases
NPV: Negative predictive value
OR: Odds ratio
PN: Patient navigator
PPV: Positive predictive value
RHS: Regional health systems
ROC: Receiver operating characteristic
SD: Standard deviation
SGH: Singapore General Hospital
